# Online Age Verification: Government Legislation, Supplier Responsibilization, and Public Perceptions

**DOI:** 10.3390/children11091068

**Published:** 2024-08-30

**Authors:** Chelsea Jarvie, Karen Renaud

**Affiliations:** Department of Computer and Information Sciences, University of Strathclyde, Glasgow G1 1XQ, UK; chelsea.jarvie@strath.ac.uk

**Keywords:** children, online harms, age verification, online service provider responsibilization

## Abstract

There are widespread concerns about the online harms to children operating online. As such, governments have enacted laws to require online service providers to deploy age verification to prevent such harms. We investigate the following three research questions regarding this topic: (1) To what extent have different governments legislated age verification controls? (2) Do governments embrace a responsibilization strategy when it comes to online age verification? and (3) How does the UK public feel about online age verification legislation? We find that governments are applying a responsibilization strategy, which has led to widespread deployment of privacy-invasive or ineffective age verification. The former violates the privacy of underage users, with the latter undermining the overarching aims of the legislation. We have also found general disengagement and a lack of trust in the government amongst the public with regards to new online age verification laws within the UK. To conclude, despite governments globally looking to put more robust online age verification mechanisms in place, there remains a general lack of privacy preservation and affordable technological solutions. Moreover, the overarching aims of the online safety and age verification legislative changes may not be satisfied due to the general public stakeholder group’s disengagement and lack of trust in their government.

## 1. Introduction

Children are increasingly operating as independent agents online, without direct supervision [1]. In this situation, the following four categories of harm can occur [2]: (1) being contacted by adults with ill intent, (2) conducting activities in adult-only spaces, (3) viewing adult-only content, and (4) commerce—the risk from online gambling, inappropriate advertising, phishing, or financial scams.

With respect to contact, Bark has reported that 8% of tweens (children aged 9–12 years old) and 10% of teens using their platform received messages classified as predatory [3] in 2023. With respect to content, research by Ofcom in 2022 showed that 60% of children aged 8–12 have their own social media account, despite the minimum age being 13 [4]. When this is combined with findings from the Children’s Commissioner in England that the top source for young people viewing pornography is X (formerly known as Twitter), then the ease at which children can access adult spaces and content online is alarming [5]. The long-term development impact on children who are exposed to adult content online was highlighted in a report published in 2016 by the National Society for the Prevention of Cruelty to Children (NSPCC, London, UK), Middlesex University, and The Children’s Commission. 

With respect to conduct, cyber bullying is prevalent, particularly on social media platforms such as YouTube, Snapchat, TikTok, and Facebook [6]. Bark has reported that 76% of teens using their platform experienced messages categorised as cyber bullying [3].

With respect to commerce, some organisations may deliberately target children with adverts. Children may find it difficult to identify adverts online [7], and it is likely that children will see adverts that are developed for adults [8,9]. As such, governments increasingly require online service providers to use an age verification mechanism as a kind of gateway which only permits adults to enter [10,11,12].

To prevent online harms to children, governments across the world have started to enact legislation to better protect children online by requiring the deployment of robust age verification mechanisms [13]. Children’s online safety groups have been advocating for the necessity of legislated online age verification for years, but progress has been slow [14]. The UK government finally introduced the UK Online Safety Act 2023 after years of discussion and heated debate [15]. The Act covers a range of different online harms such as tackling illegal and harmful content, and mandating age limits with considerable fines in place for technology companies who do not comply. The guidance states solutions must be “highly effective”, with a risk-based approach being recommended [16]. 

While the legislation is welcomed, the following two issues have emerged: the first being that it is not clear how the appropriateness of the deployed mechanism should be measured. The second related to the lack of guidance on how to achieve robust age verification [17]. In this paper, we focus on the latter, with the former being suggested as a topic of future research. Online age verification is technically challenging, and the lack of clear guidance or nomination of one or more recommended mechanisms might lead to a reality which diverges from the fundamental aims of the legislation. Some businesses are stepping in to sell solutions [18], but these are often privacy invasive [19].

There exists a gap for an age verification technology which has the following three key qualities [18]: (1) effective, (2) privacy preserving, and (3) affordable. The Age Verification Providers Association estimates that, within the next 10–15 years, the annual revenues of the age verification market across the countries, which are part of the Organisation for Economic Co-operation and Development (OECD), would be around £ 9.8 billion [20]. The age verification industry is set to become incredibly lucrative, compounded by legal requirements and the fact that there is currently no government-operated age verification solution available within the UK.

Newly enacted age verification legislation might well fail to deliver on its promise when technology companies are not being given clear guidance on how to implement online age verification. In this paper, we examine the global legal landscape for online age verification and the use of responsibilization strategies in the age verification space. Specifically, we seek to answer the following three research questions. The first two investigate the online service provider stakeholder group (the verification mechanism deployers), with the third exploring the perceptions of the UK citizenry stakeholder group (the verification mechanism users).

- Research Question 1 (RQ1): Do governments embrace a responsibilization strategy when it comes to age verification?
- Research Question 2 (RQ2): To what extent have different governments legislated age verification controls?
- Research Question 3 (RQ3): How does the UK public feel about online age verification legislation?

**Figure 1:**
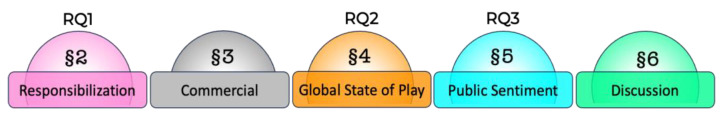
Paper Structure Mapped to Section Numbers and Research Questions.

As shown in Figure 1, we first explore the responsibilization of online service providers in Section 1 to answer RQ1. To answer RQ2, we explore the global state of play with respect to age verification in Section 3. To answer RQ3, Section 4 discusses public perceptions of UK age verification legislation. Section 5 discusses the findings of this paper with a view on potential avenues for future research, and Section 6 concludes and suggests future work.

## 2. RQ1: Responsibilization

In the UK, Former Prime Minister David Cameron launched the “Big Society” project between 2010 and 2015 [21], which focused on empowering citizens to do more for themselves and their communities [22,23]. There was a publication by the local government titled, “The Big Society: Looking after ourselves”, which aimed to advise citizens on how they could get involved in reducing crime, which would ultimately reduce police workload [22]. One of the consequences of a responsibilization strategy, such as the Big Society, is that the government then moves into a position where they are governing from a distance, as Miller and Rose have argued [24].

Responsibilization in the Western political landscape has been studied consistently since the 1990s [25]. This neoliberal transfer of responsibilities from the government to non-government organisations and citizens [26] can be motivated by a lack of government capacity and budget [27] but framed as a nudge towards citizen duty and adding public value [28]. One of the most apparent cases of responsibilization is in the healthcare space [29], where citizens are responsibilized from childhood to lead a healthy lifestyle through diet, exercise, and refraining from activities such as smoking [30]. 

In a number of countries, citizens are responsibilized to take care of their own cybersecurity [31] and parents are responsibilized to teach their children about cybersecurity [32]. Many states have websites available for citizens to get advice. Renaud et al. [33] have argued that this is not sufficient, given the complexity and specialist skills required to secure devices.

The responsibilization strategy includes the following elements:

- Provision of advice—The UK’s Cyber Strategy [34] has charged the National Cyber Security Centre (NCSC) with supporting all sectors of society to ensure that they can protect themselves from online threats. This includes the responsibility of tailoring advice for the different sectors of society.
- Responsibilization—Responsibilization hinges on advice. Governments provide such advice, with the assumption that the advice will be followed and that consequences will be accepted if something goes awry [33].
- Infrastructural services—Governments act to reduce the number of threats and harms that individuals have to deal with. For example, the UK government provides a takedown service that removes potentially harmful online content and works with large technical companies and organisations to help them to improve their security offering. Governments also push technology companies to embed security functionality into the core of digital technology. For example, the UK government is spearheading ‘Secure by Design’ legislation called Product Security and Telecommunications Infrastructure (PSTI) Act to push towards more secure architectures for computer hardware.

**Figure 2:**
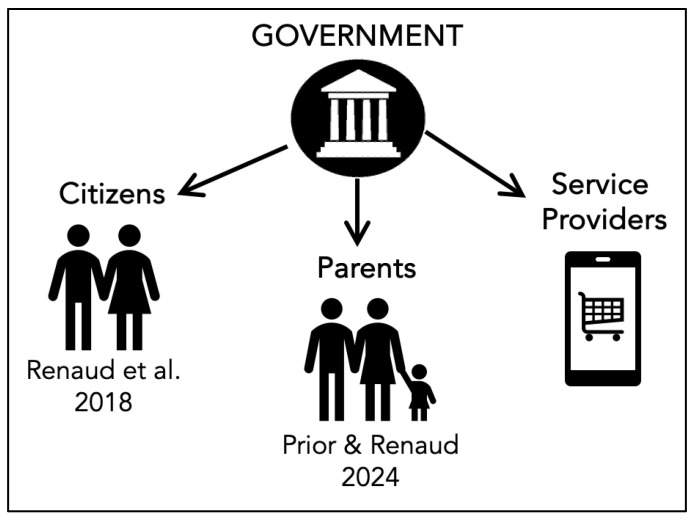
Responsibilization Types [32,33].

**Figure 3:**
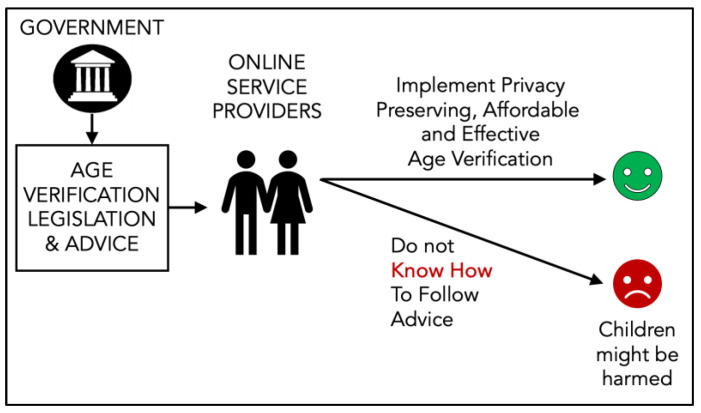
Responsibilization of Service Providers.

So far, we have spoken about ‘Individual Citizen Responsibilization’ (See Figure 2). In the context of age verification, we want to explore ‘Service Provider Responsibilization’. If online service providers are being responsibilized in the same way that citizens are, in this case, to act on their age verification responsibilities, they will have to rely purely on the proffered advice to meet legislated requirements. If they are unable to do so, children could be harmed (Figure 3). Note that age verification is a cybersecurity issue, because the mechanisms behind it assure the moderation of “availability”, which is one of the three core information security requirements (confidentiality, integrity, availability).

**Figure 4:**
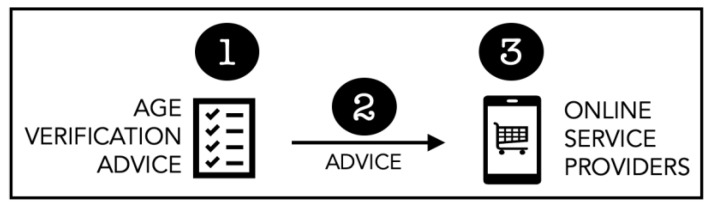
Issues with Advice.

It is worth examining a number of dimensions of advice related to age verification, as shown in Figure 4.

1. What Advice? The following should arguably be included [18]: (a) How to ensure Effectiveness—All solutions must have success requirements and a measurement strategy [35] to ensure that they do indeed act to keep children out of adult spaces. The government should provide guidance on the acceptable false positive and false negative percentages that indicate effectiveness. (b) How to verify Privacy Preservation—Reassurances from third party providers are seldom sufficient to ensure that privacy is preserved. There is a need for guidance in terms of how to ensure that the privacy of children using a mechanism is preserved if a third party supplier is used to provide age verification services. (c) Approved Age Verification Providers—Many online service providers, needing to deploy an age verification solution but not knowing how, will pay someone for their solution. The government could assist by providing a list of approved suppliers. Failing that, service providers could be certified to provide guidance to service providers choosing a third party to supply age verification.
2. Advice Delivery: There is a need to measure the effectiveness of the advice being given to service providers. Ensuring the advice is effectively communicated, accessible to a range of reading abilities, and easily understood is critical for achieving success and a high level of compliance with the law. It is crucial to minimise the risk of varying interpretations, or the risk of ignorance, if advice does not reach all stakeholders.
3. Online Service Providers: For those service providers and recipients of the advice, once it is understood what is required, there is then the issue of balancing compliance with business concerns such as affordability, effort, and expertise required. For some smaller businesses, balancing their current business models with the changing legal landscape can introduce fundamental dilemmas around how they can operate and remain profitable moving forward [18].

We carried out a scoping review of the literature to gain insights into the related research in this area, which we report on next.

### 2.1. Current Practice in Child Protection

**Figure 5:**
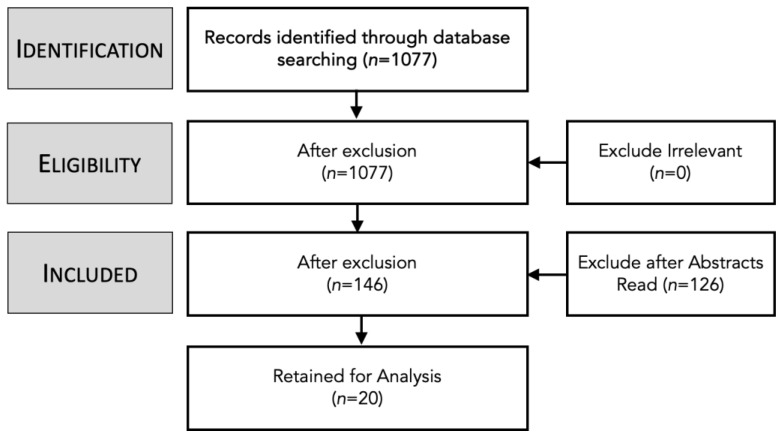
PRISMA of Scoping Study [36].

Search Process: A scoping review was carried out to ascertain the extent to which current research and the grey literature could answer the research questions posed in this paper. As shown in Table 1, a scoping study was chosen as it works best when covering a broad topic and helps identify the key concepts of the research area. The aim in doing this research was to reveal the current legal state of play regarding age verification (RQ1) and whether governments are embracing a responsibilization strategy towards online service providers. A variety of databases were used to gather relevant research including Scopus, Open Alex, and IEEE, in addition to the Google search engine for the grey literature. Material was collected for the years between 2013 and 2023. Finally, we used ChatGPT to search for any additional texts that may have been missed in previous searches. The methodology used is the approach proposed by Challen et al. [36], with the mapping and method depicted in Table 1 and the PRISMA shown in Figure 5.

Phase 1—Identification: A total of 1077 resources were found from the databases listed using the following keywords: “Government responsibilization”, Children’s Online Safety”, “Sentiment Analysis”, “Online Age Verification”, and “Online Safety Law”.

Phase 2—Screening: After the initial screening of titles, it was found that 86% of the results were not relevant due to being out of scope or context. There were a considerable number of papers rejected regarding clinical studies and healthcare.

Phase 3—Eligibility: After reviewing the abstracts of the remaining 146 papers, 75 were retained.

Phase 4—Inclusion: The 75 papers were fully read and reviewed. The final review process eliminated 55 papers as not being relevant to the scope of this study, with 20 remaining and being included. Table A2 lists the final 20 papers.

### 2.2. Synthesis of Findings 

There is a lack of evidence related to appropriate online age verification deployment. In the USA, it was found that 46% of online alcohol retailers used no age verification mechanisms [38], and 41% of tobacco retailers used the “tick box” method [39]. This issue has not gone unnoticed, and there has been significant legislative progress made by governments globally to improve the current position. However, as with any new legislation aimed to implement better guardrails around existing and embedded issues, there are a number of challenges which have yet to be fully addressed.

#### 2.2.1. Child Safety 

Every day, 170,000 children access the internet for the first time [40]. However, there is currently a race to add safeguards to the internet, which has grown and expanded without children’s online safety as a mandatory requirement [18]. Professor Byron [41] published a report in 2008 discussing the three categories of online harms that concern children; Byron labelled them the three Cs as follows: (1) Content, (2) Conduct, and (3) Contact. This was expanded in 2023 to include a fourth category—(4) Commerce [2].

- Content—Exposure to inappropriate adult content online due to the general lack of robust online age verification mechanisms on websites, apps, and particularly on social media platforms [19] is a growing concern. More than half of the 11–16-year-olds surveyed by the NSPCC had seen explicit content online [42], and Ofcom reported that 33% of British children aged 12–15 have come across sexist, racist, or discriminatory content online [43]. Recent studies have found that the age verification mechanisms employed by social media companies when users try to sign up to use the platform are significantly lacking, with children being able to circumvent seven different popular social media apps age verification mechanisms [44].
- Conduct—When learning how to navigate the internet and online platforms, teens in particular can engage in risky online conduct [45]. Sexting is a rising concern [46], but a new type of scam called sextortion has seen tragic consequences with multiple teenage suicides [47,48]. Children are also increasingly exposed to online abuse or cyber bullying [49]. Ngai et al. found that social media has become a growing problem for youth since 2005, particularly when it comes to cyber bullying [50]. Social media is not the only environment where children are at risk, the gaming ecosystem poses similar harms [51].
- Contact—The International Centre for Missing and Exploited Children state that it can take as little as 18 min for an online predator to convince a child to meet them in person [52]. The pandemic has exacerbated online safety concerns, with child abuse cases more than doubling within the first four weeks of lockdown in the US [53]. However, the benefits of the internet and the push towards online learning, particularly during the pandemic, has resulted in a trade-off with online safety being compromised [54]. As technology advances and societal behaviours change, global legal systems have been unable to adapt at the speed necessary to offer the right level of protection [55].
- Commerce—Children are certainly targeted by advertisers [56]. Much of the advertising is deceptive [57] and/or not beneficial [58]. Researchers have raised concerns about the influence that adverts can exert on children when online [59]. There are grave concerns about some kinds of advertising such as for gambling [60] and unhealthy food [61,62].

When it comes to who is responsible for the online safety of children, there are various opinions on where responsibility lies. O’Dell and Ghosh have argued that a national standard for online safety must be developed, and that schools, governments, and organisations need to strengthen their policies regarding children’s use of technology, particularly education technology [63]. However, there has also been a growing reliance on parents [32]. In countries where a general education system is provided by the government, there has always been a push towards parents supplementing their offspring’s education after school hours. In some countries, this has gone as far as schools promoting private tutoring [64]. When focusing on cyber security and children’s online safety, this has been pushed by governments as a topic that needs to be predominately taught at home, rather than within the curriculum [65]. However, as argued by Prior and Renaud, this puts parents in a difficult position and they may not have an up-to-date understanding of cyber security and cyber safety [32,65].

#### 2.2.2. Age Verification

The UK Information Commissioner’s (https://ico.org.uk, accessed on 12 August 2024) risk-based advice on age verification lists a number of verification strategies providers can employ, including self-declaration [66], a method which does not perform any robust age verification or assurance process [18]. Where regulators are empowering providers to make the right decisions, there lies a conflict of interest between business operations and profit on the one hand, with online age verification on the other. It poses the question, with the government responsibilizing providers to implement age verification as they deem necessary, of whether the introduction of laws will actually lead to a change in the way age verification is implemented? There is a risk that more providers will use the “tick box” method, which is currently one of the most popular age verification methods [18], if their individual risk assessments deem this appropriate.

Social media companies have been scrutinised for their lack of control over the age of their customers and the content that they can be shown [67]. Research from Ofcom in 2022 showed that 60% of children aged 8–12 have their own social media account, despite the minimum age being 13 [4]. This is a serious concern for the well-being of children who can be exposed to inappropriate content. In the tragic case of Molly Russell, social media platforms targeted her with inappropriate content on the topics of self-harm and suicide. The coroner concluded that the 14-year-old died as she was suffering from the “negative effects of online content”, a conclusion which sparked significant debate on what big tech companies are doing to protect children [68].

Meta Platforms Inc., Cambridge, MA, USA, owns three major social media platforms, namely Facebook, Instagram, and WhatsApp. The tech conglomerate’s platforms account for three of the top five most popular social media networks, with Facebook still holding the number one position [69]. Meta’s platforms have an age limit of 13 years old to use their services; however, in 2017, Meta released Messenger Kids for children under 13 years old to communicate with friends and family, where parents were able to monitor and control usage [70].

Meta Platforms Inc. has come under heavy fire from law makers, child safety advocates, and the UK’s data privacy regulator over both users’ privacy concerns and, in particular, their actions to protect children and their privacy while using the services. The United States Federal Trade Commission recently criticised Meta for failing to protect young users, stating that the company’s behaviour was “reckless” [71]. This stemmed from a bug found in the Messenger Kids service where the safeguard to prevent children from communicating with anyone other than friends and family was found to be flawed. There have been questions around this service and whether it complies with the Children’s Online Privacy Protection Act (COPPA) [72]. As part of this research, a review of the Messenger Kids Privacy Policy was attempted, but access would not be granted until a valid Facebook user logged in, making it challenging to review the policy without first signing up to the service.

Similarly, the privacy and safety measure for children using Meta’s Oculus Quest 2 Virtual Reality headset was questioned by the UK’s Information Commissioner’s Office (ICO). Concerns around the chat function within the app were raised, along with whether the service complied with the Age-Appropriate Design Code [71].

It would be remiss to discuss the privacy and safety issues of Meta Platforms Inc. without acknowledging the Cambridge Analytica scandal, one of the most famous privacy scandals in recent years. In 2018, it was revealed that the British firm, Cambridge Analytica, had used Facebook data to target users with political ads with the aim of influencing how they vote, with these data being used without Facebook customers being aware or providing consent [73]. Although Meta did not admit to any wrongdoing, they did pay $725 m to settle the case in the U.S. in December 2022 [73]. Given Ofcom’s statistics on the number of Facebook users under the age of 13, it is very likely that the children’s data were used inappropriately by Cambridge Analytica.

Meta have been fined by a number of EU countries for data privacy violations, including the UK [74], Italy [75], Turkey [76], and Ireland [77]. In total, as of January 2023, it is estimated that Meta has paid around $1 billion in GDPR fines due to violations in the EU [78].

TikTok has fast become a hugely popular video social media platform with a projected reach of 1.8 billion users in 2023 [79]. However, although one of the newer social platform additions to the market, TikTok has come under scrutiny for its preservation of user privacy [80], potential impact on national security [81], and its use of children’s data [82].

TikTok has stated that their app is for users over the age of 13; however, on the Google Play Store, it is marketed as 12+ years with “Parental Guidance Recommended”, which is contradictory to their policy [83,84]. TikTok verifies a user’s age by asking for their full date of birth and, where a user is less than 13 years old, they will supply them with a censored version of the platform [84]. This age verification process was deemed unsuitable by the UK’s Information Commissioners Office, who recently fined TikTok £12.7 million for violating the GDPR and misusing children’s data. The ICO believed that TikTok did not diligently verify a user’s age and take the appropriate action to remove those users who were under 13 years old [82].

## 3. Commercial Products

There is a small selection of commercial age verification solutions that vendors can pay for and have implemented into their websites. The available commercial products utilise a variety of methods to verify a user’s age. The predominant methods use database checks or photos of the user that utilise AI to determine whether the user is underage.

Yoti, a global provider, uses AI to determine the user’s age from the camera and also offers a digital ID scheme whereby a user uploads a government document and is provided with a QR code which can then be used by vendors to prove their ID. Yoti’s age verification product is the only one to be certified by the new Age Verification Regulator under the British Board of Film Classification (BBFC) age verification scheme [85]. Similar to Yoti, VerifyMyAge, Veriff, Ageify, and Luciditi use AI to estimate the age of the user [86,87,88,89], while AgeChecker.net, a U.S. provider, and Jumio, a global provider, require a user to upload a selfie with their government-issued ID. AI is then utilised to determine the age of the user [90,91].

Some vendors only accept credit cards as a means of age verification; VeriMe allows age verification of customers who want to use a debit card [92]. This is achieved via vendors obtaining debit card information, while VeriMe checks that the user’s mobile number is registered to an adult over 18. AgeChecker.net, AgeChecked, and VerifyMyAge also utilise a mobile number as a means of age verification [86,91,93]. Equifax, Experian, and Trulioo, all global products, rely on third-party database checks for age verification [94,95,96]. AgeChecked, a UK provider, are the only vendor who claim to be able to do age verification through social media, but it is unclear how this method works in practice and whether it is GDPR compliant. They also offer several other methods of verification [93]. Tencent [97] uses facial recognition to prevent children from entering their gaming platform. OneID gives customers a privacy-preserving way of verifying their age through their online banking app [98].

Some commercial products estimate the age of a user from a facial biometric. Four of the most popular tools were tested by Jung et al. [99]. They found that none performed well when it came to age determination using a static image, making them unsuitable for online age verification. Yoti claims to have a 0.08% error rate and a Mean Absolute Error of 2.09 years [100]. With current online age verification mechanisms lacking and social media companies coming under scrutiny due to safeguarding and welfare issues for children using their platforms, it is unsurprising that governments are taking action to formalise age verification. However, with this legal pressure, there remains a question on whether the technology solutions currently available meet the needs of both providers and consumers. Table A1 in the Appendix A provides an overview of the age verification mechanisms that are currently available to online service providers.

### 3.1. Responsibilization of Service Providers

There is evidence of advice provision from the government but no evidence of additional support. With respect to advice, online age verification guidance from official bodies within the UK and Europe empowers online providers to take a risk-based approach when implementing online age verification techniques [66,101]. The CNIL, France’s Data Protection Agency, emphasises the importance not only of suitable age verification technologies but the need for better cyber awareness amongst children, parents, and the wider community [101]. The move towards risk assessment, risk management, and awareness campaigns indicates that governments are indeed employing a “responsibilization” strategy when it comes to the use of age verification [102]. Organisations and communities are being given the responsibility for protecting children online through education and technical means.

Over the years, guidance around online safety has increased in line with the rise of social media and has been necessary due to the lack of legal duties placed on technology companies. Core online systems have been allowed to grow without any online safety legal framework to abide by, resulting in citizens having no choice but to take responsibility for using these online services safely [103]. When these citizens are underage, this responsibility currently falls to their parents [32].

### 3.2. Conclusion

It seems reasonable to conclude that the current approach by UK legislators reflects a responsibilization model whereby online service providers are required to comply with related legislation without much more than tailored advice. Moreover, the provided advice does not meet the minimum requirements as outlined above.

Although the legal changes are being discussed and implemented across the world, we found that there remains a consistent contention between the reality of what these mean for citizens and where responsibility truly lies, particularly when governments are choosing to implement laws and roll them out using a responsibilization strategy. This contention is discussed further in Section 3 and Section 4.

## 4. RQ2: Global State of Play

An analysis of the global legal frameworks regarding online age verification was carried out, organising the results using the United Nations Geo-scheme which splits the world into six geographic regions [104]. These regions are Europe, Asia, the Americas, Africa, Oceania, and Antarctica; however, Antarctica has not been included as it does not have a judicial system [105].

### 4.1. Europe

Significant progress is being made across the EU, by both individual countries and as a larger political union. One of the most significant EU age verification laws is the Audiovisual Media Services Directive (AVMSD). The directive came into force in 2010 and aims to ensure children are protected from harmful content within video-on-demand services; however, the interpretation of video-on-demand services has been different across the EU, and it is questionable whether social media platforms fall into scope [106]. Alongside the AVMSD, the EU has implemented several other initiatives and projects aimed at protecting children from harmful online content. The EU Kids Online project is an initiative aiming to understand how the internet poses both risks and opportunities for children. The EU Kids Online project researches topics concerning children and their interactions with the internet, including privacy, age verification, and online safety. There have been multiple research outputs of the project, including a report on the position of online age verification in use across the EU [107].

The 2016 report into the harm caused to children exposed to online adult content set in motion a flurry of activity by the UK government. The UK was on track to become the first country in the world to introduce age verification for porn sites through the Digital Economy Act 2017 [108]. However, technical difficulties relating to the implementation of these policies proved too challenging, and this was dropped in 2019 [109]. Legal proceedings began from the children’s safety activist groups against the UK government, who claimed that they had failed to protect children in the UK from the identified harms caused by exposure to adult content [110]. John Carr, an online safety consultant, and Robin Tombs, the founder of Yoti, have separately urged the UK government to make it compulsory for age verification to be completed for online pornography sites [111].

In February 2022, the UK government announced that age verification for online porn sites would become compulsory, fulfilling the promise they made five years earlier [112]. However, it is part of the Online Safety Bill, which tackles multiple online safety concerns and, similarly to the Digital Economy Bill 2017, has faced multiple challenges and delays since its inception in 2019 [113]. The Bill was due to go to House of Lords at the end of July 2022, but the Conservative government chose to delay this for at least three months while a new Prime Minister was being elected due to the resignation of Boris Johnson [113]. The Online Safety Bill was finally given Royal Ascent and became law in October 2023 [114].

Bringing together countries across the EU, the euCONSENT project aims to develop a consistent and EU-wide online age verification and parental consent system. Funded by the European Commission, the euCONSENT consortium brings together experts, companies, and governments from across the EU, the UK, and Australia to help advise and develop a solution [115].

In February 2022, the project began its first pilot with 1600 participants across five countries, namely Greece, UK, Germany, Cyprus, and Belgium. Adults, parents, and children were all included as participants and to test their age verification mechanism; children were asked to try to access three different types of websites that either needed age verification or parental consent. The methods of verification were either by using AI for age estimation, scanning a government ID or entering credit card details [116].

The euCONSENT project is ongoing, with the final pilot completed by the end of May 2022 [116]. The system was demonstrated at the euCONSENT 2022 Conference, but France’s regulator, CNIL, stated that there was no suitable age verification options which could meet all the privacy and security requirements [117]. However, doubts on the future of the project were raised by the project’s coordinator, Kostas Flokos, who called for more funding to the keep the project running [117].

Within Europe, multiple countries are beginning to take measures to better protect children online. Germany has multiple laws aimed to protect children, one being related to age verification for access to adult content and products. The Youth Protection Act was introduced in 2021, requiring media companies to appoint a Youth Protection Officer who has responsibility for ensuring the appropriate age ratings of hosted content [118]. This requirement is similar to the requirements of the GDPR, to have an appointed and named Data Protection Officer, forcing companies to take personal responsibility for data privacy and now for online youth protection as well [119].

Germany’s Commission for the Protection of Minors in the Media approved the use of Artificial Intelligence (AI) for Age Verification purposes in May 2022 [120]. As highlighted in previous analysis of age verification technology [18], the error margin for some age estimation technology can be around 2 years [100]. To combat this in Germany, the Supervisory Body stated that there must be a five year “buffer” built into the system so an 18-year-old will need the system to estimate their age to be at least 23 to gain access to online products or services [120].

In 2020, the French Parliament passed a new age verification law, which aligns the country to German law [121]. France gave adult content sites until the until the 28th of December 2022 to introduce measures or risk being geographically blocked [122]. The method of verifying a user’s age is at the discretion of the site owner; however, it has been reported that the most popular method is to ask the user for a credit card number [121].

A tick box is still the most popular age verification method, a ‘security theatre’ way of verifying a user’s age [18]. In a survey conducted in France, it was found that 44% of 11–18-year-olds lied about their age on the internet [123], demonstrating the need for more effective methods of verifying whether the user is an adult or a minor.

In July 2022, the CNIL, France’s Data Protection Authority, published recommendations for sites on compliance with age verification legislation [123]. Due to the fact that the law is light on details regarding the technical measures required to verify a user’s age, the CNIL highlighted pros and cons associated with current technology measures [123]. In addition to a review of current technology, the CNIL also placed an element of responsibility on parents to ensure that parental controls are in place on minors’ devices [124].

### 4.2. Americas

One of the most significant laws in the USA which governs online age verification is the Children’s Online Privacy Protection Act (COPPA). COPPA came into force in 1998 but was updated in 2013 to expand the definition of “personal information” to now include tags such as geolocation, videos, and photos [125]. The law requires that commercial website owners and online service providers must obtain parental consent for any users under the age of 13 before processing and collecting personal information. Operators of commercial websites and online services that are directed at children under the age of 13 must obtain verifiable parental consent before collecting personal information from children [125]. However, research by Williams et al. found that there are significant inconsistencies across the USA with regards to online age verification for both online tobacco and alcohol sales [38,39,126,127].

Canada is following a similar path to the UK with the S-210 bill, Protecting Young Persons from Exposure to Pornography Act, which aims to make age verification for pornography site compulsory [128]. However, the debate on this bill also mirrors the UK with concerns raised about privacy and security, with others concerned that it does not go far enough to include other sites such as gambling, alcohol, and weapons retailers [129].

### 4.3. Africa

Within South Africa, there are no specific online age verification laws but there is the Protection of Personal Information Act 2013 (POPIA), which is similar to the EU GDPR [130]. It has a legal framework to protect the use of children’s data, anyone under 18, and ensure the data are processed in a lawful manner as defined within the law. There is no legal requirement for age verification under this law [131].

### 4.4. Asia

In 2019, the Cyberspace Administration of China (CAC, Beijing, China) released the Provisions on Cyber Protection of Personal Information of Children (PCPPIC) which outlines a variety of additional protection for children’s privacy and security online. There are many similarities to the UK’s GDPR and the USA’s COPPA; interestingly, where it differs is that China refers to anyone under the age of 14 as a minor, significantly younger than 18 in the UK and 21 in the USA [132].

China has taken a strict approach to limiting the amount of time minors can spend playing games online as well as how much money they can spend. The State Administration of Press and Publications (SAPP) now requires users to enter their real name on games, which can be checked against a database to verify the identity and age of all online game users [133].

### 4.5. Oceania

Following a report published in 2020 by the House of Representatives Standing Committee on Social Policy and Legal Affairs titled, “Protecting the age of innocence” [134], Australia trialled age verification for alcohol, with gambling and adult sites next in line to have the measures introduced [135]. However, the technology to underpin online age verification failed, requiring ID to be checked physically at delivery [136].

In June 2021, the Australian government launched a consultation with the aim of developing a roadmap for introducing age verification for online pornography sites. As part of this work, the government asked for evidence from the industry about the privacy and security risks, the current technology available, and the impact online pornography has on youths [137]. The findings from this consultation with a road map for the future was published in March 2023.

The Online Safety Declaration 2022, part of the Online Safety Act, made it a legal requirement for online platforms to have age verification mechanisms in place to prevent minors from accessing adult content [138]. In response to this, Google proposed verifying users are over 18 through passport or driving licence verification [139].

## 5. RQ3: General Public Perceptions and Sentiment

The final stakeholder group with regards to the introduction of new online age verification laws are the general public who will be interacting daily with the online verification mechanisms. To gain an insight into the general perceptions of this stakeholder group, we carried out sentiment analysis on the UK population around the introduction of the UK’s Online Safety Act. We reviewed YouTube and Reddit comments which related to discussions and videos surrounding these legal changes, as discussed in Section 3.1.

Using the key phrase, “UK Online Safety Bill”, both Reddit and YouTube were searched for relevant videos and threads in July 2023. In total, three Subreddits and six YouTube videos were selected for inclusion, and the comments were analysed. The three Subreddits were the only threads with over 35 comments each, and the six YouTube videos had over 3000 views per video, with the average total views across the selected videos totalling 13,370. The posts and comments spanned from October 2023 to July 2023.

All three Subreddits combined had a total of 612 comments, which were scraped using PRAW and analysed using TextBlob to give a polarity and subjectivity score to each comment. There was a total of 279 YouTube comments scraped using Octoparse and sentiment analysis was carried out using TextBlob.

The polarity scale ranges from −1 to 1, with a score of −1 being negative and 1 being classified as positive. With respect to subjectivity, a high score would indicate the comment is highly opinionated rather than factual, and a low score would be more likely to be based more on fact [140].

**Figure 6:**
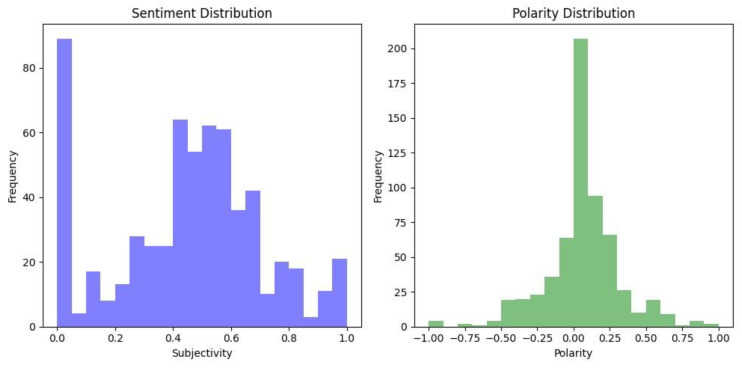
Reddit Sentiment Analysis.

**Figure 7:**
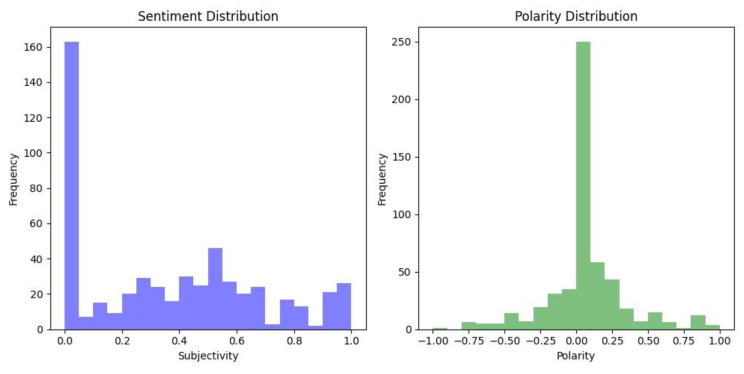
YouTube Sentiment Analysis.

Overall, the sentiment rated as fairly neutral as can be seen in Figure 6 and Figure 7. However, in reading the comments, many users raised concerns about the Online Safety Bill’s legal stipulation that messaging services must be able to decrypt user messages in a bid to better uncover the sharing of pornographic images of children [15]. Several users were concerned about an invasion of their own privacy, with some stating that they would use a VPN if encrypted services were to be blocked in the UK.

### 5.1. Reddit

In Reddit discussions where adults would need to identify themselves for online age verification purposes, one particular user commented that “It would make the whole use of internet invasive, risky and unsafe. When minors need to be protected, then better not allow them on the internet at all; like drinking, driving, using machinery”. Another user with similar personal privacy concerns commented “That sounds like they want to know where you go online. Id rather have some curiouse children see genitals than lose more and more freedom.”

Of the more negative Reddit comments, which were rated as highly opinionated and scored close to −1 for polarity, related to the discussion that the introduction of the Online Safety Bill may mean encrypted messaging apps could remove their service from the UK and access to Wikipedia may be blocked, the following are notable:

1. “Why walk at all? Continue giving the service, fully encrypted. At worst UK blocks it, which would still allow users to access via VPN”.
2. “EXPLETIVE stupid Tory government. We’ve all got access to vpns anyway”.
3. “Let me tell you, there’ll be a EXPLETIVE riot if they try to take away Wikipedia”.

When reviewing the Reddit comments deemed as neutral or with positive polarity, many of them were replies to other comments with little details, and others read as quite negative about the bill and highly opinionated, which would suggest incorrect analysis by TextBlob. An example is as follows: “So really it’s just a way for the government to better track individuals browsing activities with age verification being the excuse”.

### 5.2. YouTube

Similarly to Reddit, the YouTube comments rated as positive and factual were mainly generic comments such as “Good” or comments regarding the presenters’ appearance rather than the content of the discussion. One user did comment: “Im sure everyone will sleep a lot better when this bill is passed”. But the comments TextBlob rated similar to this one are as follows:

1. “Won’t be able to say what you like, and won’t be a to protest about it if they get their way”.
2. “Authoritarian goverment at his best, but the anglos where always kind of”.
3. “ministry of truth brilliant”.

Of the comments rated as negative and highly opinionated, there is a general feeling of distrust towards the government:

1. “This bill is extremely dangerous and must be scrapped”.
2. “The scope for abuse of this bill is vast. It is dangerous and must be scrapped”.
3. “Is the post office now going to open everyone’s mail to check whether or not people are exchanging illegal pictures or saying dangerous things?”

This distrust could see more general internet users exploring the use of the dark web for anonymity, with Kovalchuk et al. seeing an increase in dark web usage during the 2020 COVID-19 pandemic [141].

There are several comments where users state that they believe parents should be doing more to protect children online, instead of the government having to introduce laws to protect children. When discussing the measures being put in place by the bill, some users were unhappy at the balance being struck between children’s safety and the privacy of online users, as can be seen from the following:

1. “Isn’t it the parent’s responsibility?!? Can I still write my opinion of Islam or will I go to jail now”?
2. “Parents couldn’t control their kids, now the GOVERNMENT HAS TO BABYSIT US? Apps are the reason why I didn’t fall into depression”
3. “Maybe the parents need to do some parenting”?
4. “I mean the reality is that its up to the parents to keep their children protected, rather than an ever-growing list of stringent, restrictive changes to everyone elses life to compensate for it, which is ultimately what these things end up becoming”

There was an overarching feeling of unease and a lack of trust in the UK government. This could hinder the success of any responsibilization strategy, which is what the legislation is attempting to achieve.

UK citizens post-pandemic do not trust their government [142]. Indeed, the 2024 Edelman Trust Barometer [142] reports that the UK government is only trusted by 30% of the public. That being so, they would prefer not to put their faith in the government’s efforts to protect their children. They prefer to embrace that responsibility themselves. This might explain why parents are currently embracing responsibilization despite their lack of cybersecurity knowledge [32].

## 6. Discussion

This aims of this study were broad and covered the global legal position of online age verification, with a more detailed analysis around the UK due to the regulations being more mature. As we analysed the findings across the three research questions, it became clear that there are several fundamental concerns around stakeholder engagement, advice and guidance, and societal views that could all impact the aim of the legislation, which is to keep children safe online from harmful content. Returning to each of the research questions set out in our introduction, we note the following:

### 6.1. RQ1: Do Governments Embrace a Responsibilization Strategy When It Comes to Age Verification?

Where age verification has become mandatory, a responsibilization strategy is being deployed. The UK is further ahead in the global legal landscape and is advising organisations to take a risk-based approach and decide for themselves the most appropriate way to ensure that their service is either safe for or is not accessed by underage users. A responsibilization strategy in this context may not prove to be the best method to achieve the overarching aims of the age verification legislation.

Current online age verification practices are inadequate in terms of privacy preservation, affordability, and effectiveness, because a clear official mandate with guidance is lacking. Unfortunately, while internet-enabled core daily services have become embedded within society, this is occurring without the required controls to keep children safe when online. Although the legislation is a step in the right direction, without robust guidance effective technical solutions and engagement and collaboration with the affected stakeholders, the risk of online harm to children will not be significantly reduced.

Critics of the age verification legal advancements have cited surveillance concerns. When we consider this alongside the comments discovered during our sentiment analysis, we believe that the impact of online age verification laws may not live up to expectations; this is discussed further in Section 6.3.

### 6.2. RQ2: To What Extent Have Different Governments Legislated Age Verification Controls?

While governments have made an effort to start discussions to better protect children online from harmful content, or from accessing and buying adult products or services, the legal frameworks are still in their infancy. With some countries further ahead in the implementation of laws to mandate age verification, some are still developing roadmaps, while others seem to have no public plans to address this issue.

With regards to protecting children’s data, there are global legal frameworks in every continent for this. Children’s data privacy as a legislative topic is further ahead than age verification worldwide. When focusing on age verification, Western countries, particularly Europe, are at the forefront of tackling this and are working very collaboratively.

Given that the internet is borderless, we suggest that more collaboration is needed between the geographic regions, similar to the euCONSENT project and The Global Online Safety Regulators Network but providing more public engagement, research findings, or opportunities for collaboration.

This collaborative approach must take into consideration, all viewpoints, including the impact on privacy that online safety measures introduce. In order to make the internet a safer place for children, people will need to sacrifice an element of their anonymity online, particularly adults looking to access age-restricted sites and content.

### 6.3. RQ3: How Does the UK Public Feel About Online Age Verification Legislation?

When reviewing the stakeholders’ roles and interests around the introduction of online safety legislation and mandatory online age verification, charities, researchers, and children’s online safety advocate groups have celebrated the implementation of the Online Safety Act. However, in sharp contrast, a lack of trust in the UK government became clear from the Reddit and YouTube comments that we analysed. There was a sense amongst the general public stakeholder group that the government is not ultimately responsible for children’s online safety; their parents are.

Generally, many internet users were concerned about their online anonymity being jeopardised in the interest of children’s safety. When citizens do not trust their government, all government actions can be viewed with suspicion. A recent citizen survey in England found that 73% of respondents did not trust the UK government to make decisions to improve their lives [143].

The Institute for Public Policy Research (IPPR) has warned that this steady decline in trust can lead to disengagement, populism, and polarisation of our society [144]. The consequences of distrust will in turn have a knock-on effect, where governments are trying to make change to deal with long-term problems such as climate change and inequality and, we believe, the topic of online safety. Should the general public reject online age verification mechanisms, this may impact the businesses who are legally required to implement such technologies and could in turn hinder the progression of better online safety controls.

### 6.4. Practical Implications

The findings across the three research questions suggest that the aims of age verification legislation may not be met for a number of reasons. Firstly, the needs of all stakeholders do not appear to have been satisfied, and greater efforts are needed from the government to ensure that stakeholders are consulted and appropriately informed. This can help promote and secure the success of the legislation in the longer term.

1. Service Providers: Clearer guidance for service providers who are being impacted by the legislation. This is needed to prevent a fragmented approach to compliance. Although a risk-based approach can be appropriate, it is also crucial to give the context and structure by which these risks need to be evaluated to ensure consistency across Service Providers.
2. Citizens: Sentiment analysis suggests that there are differing views and scepticism towards the new-age verification legislation. With this large stakeholder group, it is critical to ensure that the intentions of any new legislation and the wider benefits to society are effectively communicated and understood. However, scepticism towards the government is a wider societal issue which will not only affect the roll out of age verification regulations but fundamental societal change and thus must be addressed.

### 6.5. Limitations

This study provides a snapshot of the global legal landscape. However, there are still a number of limitations which may have impacted the results.

We searched extensively for information about what other countries are doing in terms of age verification. However, we used English terms only. It would be beneficial to do a more wide-ranging search with a variety of different languages to gain more results from non-English-speaking countries.

The sentiment analysis used a number of keywords to search for comments on Reddit and YouTube; however, this could have been expanded to include other societal groups. To gain further insight into the UK public’s view of the changing legal landscape, a survey could have reached a more diverse audience. In particular, it would be interesting to analyse the views of parents while also seeking to understand if there are different views based on age group or gender.

Furthermore, although this research included the global legal state of play with regards to age verification, the sentiment analysis only took the UK into account. The UK has been at the forefront of online age verification law, which meant there were more data available to analyse during this study. However, given the different cultures and legal and political landscapes across the world, a global sentiment analysis study would produce a global insight.

## 7. Conclusions and Future Work

We present an overview of the global position of online age verification, which shows that there is a significant understanding that better controls are required to protect children online. Legislative progress is being made, with projects and working groups collaborating to tackle the issues. However, with regards to the deployment of these new-age verification controls, there remains a question on how effective the legislation will be when responsibility for how to satisfy legislation is assigned without the adequate guidance and support.

Similarly, without technological options which meet the legislative requirements as well as the needs of providers and consumers, there exists a risk that the implementation of online age verification controls will fail to truly deliver greater protection for children. Measuring the effectiveness of the methods that service providers opt to use for online age verification is an area of future research and development which could prove beneficial to the advancement of online age verification controls.

This is not an easy issue to rectify because children’s online safety is not part of the internet’s core design. As we know from the fields of privacy and security, it is essential to build these requirements in at the design phase; therefore, “safe by design” requirements should be part of the internet service’s design moving forward.

The findings from our research and analysis may indicate fragmentation in society which could prove challenging for the implementation of any online safety law and would be an interesting area of future research. In particular, investigating whether people are more likely to use dark web services and communication channels in order to retain their privacy.

In terms of future work, further analysis into the global sentiment of both the online service providers who are legally obliged to comply with new online safety stipulations and the global general public may show variances in opinion and deliver insights into how these laws may be more effective.

Similarly, building upon the research carried out by Renaud and Prior [65], further investigations to discover the views of parents who are being responsibilized to discover their concerns is essential. Understanding the views, opinions, and expectations of all societal groups with regards to online age verification and safety could help uncover what is needed at a strategic level for the internet to become a safer place for children to learn, socialise, and play online.

## Figures and Tables

**Table 1:** Methods of identifying and collating research evidence [37].

Method	Explanation	Purpose	Breadth	Depth of Process
Systematic Review	Carried out to produce an overview of primary studies with a specific set of objectives. It is conducted in such a way that reproducibility is fostered	Summarise a body of research in a particular domain	Specific question	Meticulously documented in-depth searching for studies relevant to specified question
Scoping Review	Overview of key concepts underpinning a particular research domain	Uncover research activity and reveal gaps in research	Broad Topic	Identify boundaries of research in a domain
Evidence Mappinv	The systematic organisation and illustration of a broad field of research evidence	Making a body of research easily accessible	Broad Topic	Providing a description of the area being studied

## Data Availability

Data is available on request. The data are not publicly available due to privacy or ethical.

## Appendix A

**Table A1:** Age Verification Products (details based on website check in July 2024).

Solution	Checks	Price
	WHAT YOU KNOW	
Renaud and Maguire [19]	Knowledge and ability to identify photos of historical figures	N/A
	WHAT YOU ARE	
Yoti [85]	Picture (AI)	25p per verification
Verify my Age [86]	Video (AI)	45p per verification (eBay)
OneID [98]	Picture (AI)	from 16p per verification
Veriff [87]	Picture (AI)	80 cents per verification plus $49 monthly fee
Ageify [89]	Picture (AI)	Basic plan $3.99 per month (Shopify)
	WHAT YOU HOLD	
Yoti [85]	Government ID	25p per verification
	Phone Number	
Verify my Age [86]	Third Party Database Check	45p per verification (eBay)
	Government ID	
	Credit Card Check	
	Phone Check	
VeriMe [92]	Phone Number Check (if using debit card)	Unknown
OneID [98]	Online Banking	16p per verification
AgeChecker [91]	Third Party Database Check	$25 per month plus 50 cents per verified user
	Phone Number Check	
AgeChecked [93]	Driving Licence	Unknown
	Phone Number Check	
	Social Media	
	Payment Card	
	Address Search	
Trullioo [96]	Government ID	Unknown
	Third Party Database Check	
Melissa [145]	Address Check	Unknown
Equifax [94]	Third Party Database Check	Unknown
Experian [95]	Third Party Database Check	Unknown
	WHAT YOU HOLD & ARE	
AgeChecker [91]	Selfie with ID (AI)	$25 per month plus 50 cents per verified user
Jumio [90]	Selfie with ID (AI)	Unknown
Tencent [97]	ID Card + Facial Recognition	Unknown

N/A—Unknown.

**Table A2:** Scoping Study Included Papers.

Author(s)	Title
Eric W.T. Ngai, Spencer S. C. Tao, Karen Ka-Leung Moon [50]	Social media research: Theories, constructs, and conceptual frameworks
D. Andrews; S. Alathur; N. Chetty; V. Kumar [54]	Child Online Safety in Indian Context
H. Pozniak [53]	The child safety protocol: In dark corners of the internet, there have been horrific consequences to children living more online during the coronavirus lockdown. Are tech giants doing enough to protect them? And will greater privacy measures allow abuse to go unchecked?
B. E. Cartwright [55]	Cyberbullying and cyber law
A. Faraz; J. Mounsef; A. Raza; S. Willis [51]	Child Safety and Protection in the Online Gaming Ecosystem
O. Kovalchuk; M. Masonkova; S. Banakh [141]	The Dark Web Worldwide 2020: Anonymous vs Safety
M. Gaborov; M. Kavalic; D. Karuovic; D. Glušac; M. Nikolic [49]	The Impact of Internet Usage on Pupils Internet Safety in Primary and Secondary School
R. Farthing; K. Michael; R. Abbas; G. Smith-Nunes [40]	Age Appropriate Digital Services for Young People: Major Reforms
L. Pasquale; P. Zippo; C. Curley; B. O’Neill; M. Mongiello [44]	Digital Age of Consent and Age Verification: Can They Protect Children?
T. O’Dell; A. K. Ghosh [63]	Online Threats vs. Mitigation Efforts: Keeping Children Safe in the Era of Online Learning
C. Doherty [64]	Responsibilising parents: the nudge towards shadow tutoring
K. Renaud [33]	Is the responsibilization of the cyber security risk reasonable and judicious?
M. Lister [21]	Citizens, doing it for themselves? The big society and government through community
K. Renaud [31]	Cyber Security Responsibilization: An Evaluation of the Intervention Approaches Adopted by the Five Eyes Countries and China
R. Williams [127]	Internet little cigar and cigarillo vendors: Surveillance of sales and marketing practices via website content analysis
R.S. Williams [146]	Age verification and online sales of little cigars and cigarillos to minors
C.J. Uittenbroek [27]	Everybody should contribute, but not too much: Perceptions of local governments on citizen responsibilisation in climate change adaptation in the Netherlands
